# Strawberry notch homolog 2 regulates the response to interleukin-6 in the central nervous system

**DOI:** 10.1186/s12974-022-02475-1

**Published:** 2022-05-27

**Authors:** Taylor E. Syme, Magdalena Grill, Emina Hayashida, Barney Viengkhou, Iain L. Campbell, Markus J. Hofer

**Affiliations:** 1 School of Life and Environmental Sciences and the Charles Perkins Centre, The University of Sydney, Sydney, NSW 2006 Australia; 2 Division of Pharmacology, Otto Loewi Research Center, Medical University of Graz, 8010 Graz, Austria; 3 Present Address: Division of Phoniatrics, Department of Otorhinolaryngology, Medical University of Graz, 8036 Graz, Austria

**Keywords:** Strawberry notch homolog 2, SBNO2, Interleukin-6, Central nervous system, Neuroinflammation

## Abstract

**Background:**

The cytokine interleukin-6 (IL-6) modulates a variety of inflammatory processes and, context depending, can mediate either pro- or anti-inflammatory effects. Excessive IL-6 signalling in the brain is associated with chronic inflammation resulting in neurodegeneration. Strawberry notch homolog 2 (*Sbno2*) is an IL-6-regulated gene whose function is largely unknown. Here we aimed to address this issue by investigating the impact of *Sbno2* disruption in mice with IL-6-mediated neuroinflammation.

**Methods:**

Mice with germline disruption of *Sbno2* (*Sbno2*^−/−^) were generated and crossed with transgenic mice with chronic astrocyte production of IL-6 (GFAP-IL6). Phenotypic, molecular and transcriptomic analyses were performed on tissues and primary cell cultures to clarify the role of SBNO2 in IL-6-mediated neuroinflammation.

**Results:**

We found *Sbno2*^−/−^ mice to be viable and overtly normal. By contrast GFAP-IL6 × *Sbno2*^−/−^ mice had more severe disease compared with GFAP-IL6 mice. This was evidenced by exacerbated neuroinflammation and neurodegeneration and enhanced IL-6-responsive gene expression. Cell culture experiments on primary astrocytes from *Sbno2*^*−/−*^ mice further showed elevated and sustained transcript levels of a number of IL-6 stimulated genes. Notably, despite enhanced disease in vivo and gene expression both in vivo and in vitro, IL-6-stimulated gp130 pathway activation was reduced when *Sbno2* is disrupted.

**Conclusion:**

Based on these results, we propose a role for SBNO2 as a novel negative feedback regulator of IL-6 that restrains the excessive inflammatory actions of this cytokine in the brain.

**Supplementary Information:**

The online version contains supplementary material available at 10.1186/s12974-022-02475-1.

## Background

Interleukin-6 (IL-6) is a pleiotropic cytokine produced in a variety of inflammatory processes. It has pro- and anti-inflammatory functions in highly context-dependent roles [1]. In the central nervous system (CNS), IL-6 is elevated after acute insults such as infection and injury, where it is thought to have a necessary and beneficial role in their resolution [2–4]. However, IL-6 is also a key cytokine in the pathogenesis of several chronic CNS diseases including several autoimmune and neurodegenerative diseases [5–10].

Virtually all resident cells in the CNS as well as infiltrating immune cells may produce IL-6 [11, 12]. Further, all cells can respond to IL-6 via a soluble IL-6 receptor (sIL-6R) and the ubiquitous membrane-bound co-receptor, gp130. In addition, a small number of cell types, such as microglia, neutrophils and CD4+ T-cells, express a membrane-bound IL-6 receptor (IL-6R). Signalling via the sIL-6R (known as “trans-signalling”) is thought to be responsible for the pro-inflammatory and detrimental effects of IL-6, while signalling via the IL-6R (known as “classical signalling”) is thought to be responsible for the anti-inflammatory effects of IL-6 [1, 13]. The JAK/STAT pathway is the main driver of the transcriptional response to IL-6, with STAT3 playing a dominant role and STAT1 a relatively minor one [13–15].

A biologically relevant model in which to study the effects of IL-6 in the CNS is the GFAP-IL6 transgenic mouse, in which IL-6 is chronically produced by astrocytes at pathophysiological levels [16–19]. This model exhibits a spontaneous, progressive neurodegeneration with glial and vascular involvement replicating many aspects of the structural and functional neuropathology found in human neurodegenerative and neuroinflammatory disorders. IL-6 transgene expression is highest in the cerebellum (specifically, in the Bergmann glia), thalamus and brain stem, but is relatively low in other regions [20]. Pathological changes in the brain overlap with transgene dose and animal age.

Despite the almost ubiquitous presence of IL-6 during inflammation, much is still to be learned regarding the mechanisms modulating its actions. We initially identified Strawberry notch homolog 2 (*Sbno2*) in a screen of genes highly upregulated in vitro in murine astrocytes and microglia in response to IL-6 trans-signalling. *Sbno2* is one of two mammalian paralogs belonging to the poorly described ‘strawberry notch family’ of conserved, nuclear, putative helicases. Family members are involved in the modulation of gene expression and have primarily been described in the context of development [21–26]. Sno in *Drosophila* and murine SBNO1 localise to the nucleus and positively regulate *Delta* and *Cdx2* expression, respectively, through processes involving the dissociation of transcriptional regulators from the genome [21, 24]. Furthermore, murine SBNO1 and *Arabidopsis* homolog FORGETTER1 associate with chromatin remodelling factors [24, 26].

Mammalian *Sbno2* is highly regulated in differentiated adult cells. We demonstrated that *Sbno2* is upregulated in murine astrocytes in vitro in response to IL-6 and other cytokines, and is highly upregulated in the murine brain in response to endotoxin-induced systemic inflammation [27]. It has been implicated in the anti-inflammatory response with expression being stimulated by IL-10 via STAT3 in murine bone marrow derived macrophages and was reported to inhibit NF-κB-mediated transcription [28]. Separately, a role was described for SBNO2 in murine osteogenesis, in which it positively regulates *Dcstamp* expression by removing the repressor TAL1 from the *Dcstamp* promoter, allowing subsequent binding of the activator MITF [25]. Importantly, these two reports on murine *Sbno2* differ on whether its deletion is embryonically lethal. However, there is at least one report of a human loss-of-function mutation of *Sbno2* causing disease and premature death [29]. Incidentally, human *Sbno2* has been associated with several CNS disorders, including schizophrenia [30], Alzheimer’s disease [31], stroke [32] and traumatic brain injury [33].

To further understand the role of SBNO2 in the CNS and, in particular, in IL-6-mediated neurological disease, we generated a new mouse model with disruption of *Sbno2* and crossed these mice with the GFAP-IL6 transgenic mice. Our results show that SBNO2 constrains the detrimental actions of IL-6 in the CNS, which is associated with an increase in IL-6 responsive genes and related ontologies, indicating that SBNO2 is a novel regulator providing negative feedback in the response to IL-6 in vivo.

## Materials and methods

### Animals

**Fig. 1:**
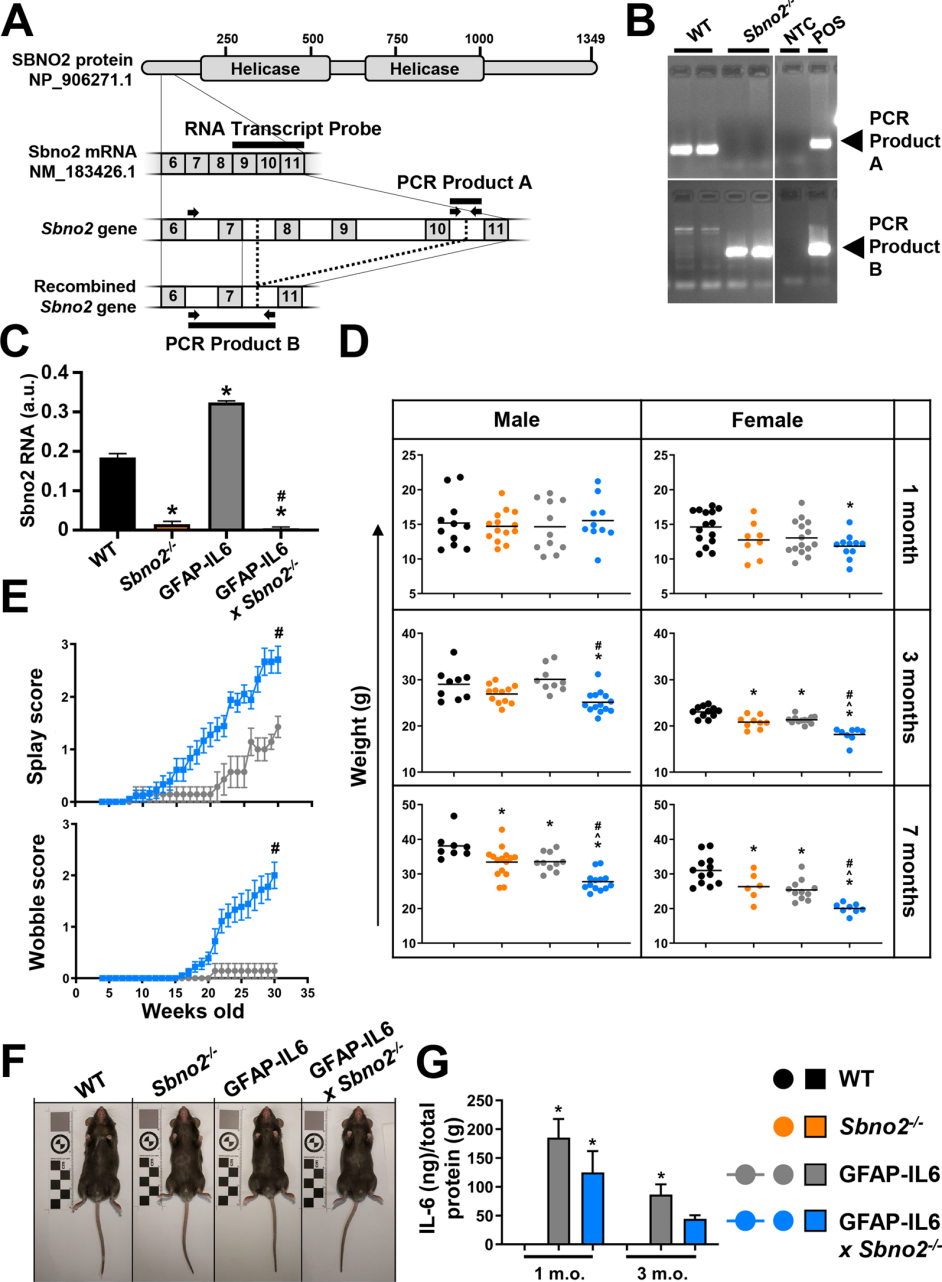
Characterisation of *Sbno2*^*−/−*^ and GFAP-IL6 × *Sbno2*^*−/−*^ mice. **A** Exons 8 to 10 of *Sbno2* were flanked with loxP sites (dotted line) and were excised by Cre recombinase. PCR primers (arrows) generated a 108 bp product from the WT locus (PCR Product A) or a 355 bp product from the recombined locus (PCR Product B). A probe against exons 9 to 11 (RNA transcript probe) was used to detect Sbno2 transcript by RPA. **B** PCR using tail DNA indicated that the unrecombined *Sbno2* locus (PCR product A) was present in WT mice whereas the recombined locus (PCR product B) was present in *Sbno2*^*−/−*^ mice. NTC, no template control; POS, positive control. **C** RNA extracted from the cerebellum was used in RPA to measure Sbno2 transcript. Transcript was detectable in WT mice and was increased in GFAP-IL6 mice, but was not detectable in *Sbno2*^*−/−*^ or GFAP-IL6 × *Sbno2*^*−/−*^ mice (*n* = 3 per genotype). **D** Male or female mice were weighed at 1, 3 and 7 months of age (*n* = 6–15 per genotype). **E** Clinical scoring of gait in GFAP-IL6 and GFAP-IL6 × *Sbno2*^*−/−*^ measured development of ataxia (*n* = 7–18 per genotype). Weights at 30 weeks of age were compared by Mann–Whitney U test and presented as the mean ± SEM. The mean age of onset was 23 weeks for GFAP-IL6 mice and 18 weeks for GFAP-IL6 × *Sbno2*^*−/−*^ mice, although this was not significantly different. **F** All mice appeared physically normal at all ages examined. Representative images of 3-month-old male mice shown. **G** IL-6 could not be detected by ELISA in lysates of cerebellum from 1- or 3-month-old WT mice but was present in GFAP-IL6 and GFAP-IL6 × *Sbno2*^*−/−*^ mice (*n* = 3 per genotype). *, *p* < 0.05 compared with WT; ^, *p* < 0.05 compared with *Sbno2*^*−/−*^; #, *p* < 0.05 compared with GFAP-IL6

All mice were on the C57BL/6 background, were maintained in the Molecular Bioscience (building G08) animal house facility at the University of Sydney under specific-pathogen-free conditions and received food and water ad libitum. The generation of *Sbno2* “floxed” mutant mice was performed by Ozgene Pty Ltd, Australia. Briefly, a conditional allele of *Sbno2* was created by flanking exons 8 to 10 (corresponding to Genbank accession NM_183426.1) with loxP sites (Fig. 1A). Gene targeting was performed in C57BL/6 embryonic stem cells and the neomycin cassette introduced during this process was removed. The *Sbno2*-floxed mice were then crossed with male B6.Cg-Tg(Gfap-cre)73.12Mvs/J mice which express the enzyme Cre recombinase in gametes. Cre-mediated recombination of the floxed exons was designed to cause a translational frame shift, introducing an early stop codon in exon 11, resulting in nullizygous (*Sbno2*^*−/−*^) mutant mice. Furthermore, to generate GFAP-IL6 × *Sbno2*^*−/−*^ mice, the Sbno2^−/−^ mice were crossed with GFAP-IL6 mice (originally obtained from the Scripps Research Institute, La Jolla, CA, USA, where they were developed by I. L. Campbell), which chronically produce IL-6 from astrocytes in the CNS at pathophysiological levels [16]. Genotyping primer pairs used to detect *Sbno2*^+*/*+^ (wildtype, WT) or floxed *Sbno2* sequence were 5ʹ-GACTGCCAGCTGAATCCAAAC-3ʹ (forward) and 5ʹ CCCACAAGCCTCCATTTTCC-3ʹ (reverse); to detect recombined sequence, 5ʹ-GGGAAAACTGAGACCCCACC-3ʹ (forward) and the same reverse as for the WT/floxed sequence. Clinical scoring was based on the Table 1 of Metten et al*.* 2004 [34] with the following modifications: individual mice for observation were removed from home cage and placed into a separate cage with no housing; mice were immediately observed by eye for 30 s and “splay” and “wobble” scores recorded; scoring was always performed by the same person; a wobble score of “1” was only recorded when the mouse wobbled while moving; “onset” was determined by a score above 0. For euthanasia and tissue collection, mice were subjected to deep anaesthesia by intraperitoneal injection of ketamine at 75 mg/kg in combination with xylazine (Troy Laboratories) at 10 mg/kg (10 µl/g body weight) before perfusing with approximately 20 mL of ice-cold phosphate buffered saline (PBS) (137 mM NaCl, 2.7 mM KCl, 10 mM Na2HPO4, 1.8 mM KH2PO4) using a gravity-fed set-up (approximate pressure 120 mmHg, approximate flow rate 3 mL/min). Unless otherwise stated, sex was not controlled for. Tissue collected for histochemistry and immunohistochemistry was placed into PBS-buffered 4% (w/v) formaldehyde overnight. The tissue was processed to extract RNA and protein as previously described [13].

### Histochemistry and immunohistochemistry

Paraffin sections were prepared and routine histochemistry (hematoxylin and eosin (H&E); luxol fast blue and cresyl violet (LFB)) was performed as previously described [35]. For immunohistochemistry (IHC), tissue was deparaffinised in xylene and rehydrated in a graded ethanol series. Antigen retrieval was performed at sub-boiling temperature as follows: for GFAP, in 25 mM Tris (pH 9.0) for 25 min; for IBA1, in 25 mM Tris–HCl (pH 8.0), 5 mM ethylenediaminetetraacetic acid, 0.05% (w/v) sodium dodecyl sulfate for 45 min; for CD3, in 10 mM citric acid for 45 min. After incubation, slides were cooled in retrieval buffer to approximately room temperature on ice then briefly washed in PBS. Endogenous peroxidases were blocked with 0.3% (w/v) hydrogen peroxide in PBS for 10 min then briefly washed. Tissue sections were encircled using a ‘pap’ pen (Sigma-Aldrich) to form a hydrophobic barrier, blocked with IHC blocking buffer (1% (v/v) normal goat serum in PBS with 0.1% (v/v) Triton-X100) at room temperature for 30 min in a humidified chamber and then incubated with primary antibody diluted in IHC blocking buffer at 4 °C overnight in a humidified chamber (GFAP, 1:2,000, Dako #Z0334; IBA1, 1:2,000, Wako #019-19741; CD3, 1:200, Abcam #16669). The next day, slides were incubated with biotinylated secondary antibody (Rabbit IgG-Biotin, 1:200, Vector Labs #BA1000) for 30 min in a humidified chamber, followed by incubation with avidin-conjugated horse radish peroxidase (Vector Labs) for 20 min in a darkened humidified chamber. Immunocomplexed protein signal was detected using 3,3'-diaminobenzidine HRP substrate (Vector Labs) then counterstained with hematoxylin. Sections were dehydrated in a graded ethanol series, then cleared with xylene before coverslips were mounted using dibutylphthalate polystyrene xylene mountant (Sigma-Aldrich). Bright field images of sections were acquired using a ZEISS Axio Scan.Z1 Slide Scanner and ZEISS Zen Blue software. For quantification, images were deconvoluted using ImageJ to remove the signal from hematoxylin counterstaining. Densitometric analysis of 3,3'-diaminobenzidine (DAB) intensity from GFAP and IBA1 staining in the cerebellar white matter, cerebellar molecular layer and cerebellar granular layer was performed using ImageJ. Per section, the average signal intensity for each region was quantified from 5 locations of a uniform area. Cell counting of CD3-positive cells in the cerebellum was performed manually and was normalised to the measured area (arbitrary units).

### IL-6 enzyme-linked immunosorbent assay (ELISA)

Levels of IL-6 in cerebellar and forebrain lysates were detected and quantified using the IL-6 Mouse Uncoated ELISA Kit according to the manufacturer’s instructions (ThermoFisher Scientific #88-7064-88). The plate was scanned with a FLUOstar Omega microplate reader.

### Quantitative real-time PCR (qPCR)

DNA was removed from 1 ug RNA using RQ1 DNaseI (M6101, Promega) following manufacturer’s instructions. Following DNaseI denaturation, cDNA was synthesised using the RevertAid RT Reverse Transcription Kit (K1691, Thermo Fisher Scientific) according to manufacturer’s instructions. cDNA was subsequently stored at − 20 °C. To quantify the expression of *Sbno2* transcript, 10 ng cDNA was added to 0.4 μM forward and reverse primers 5ʹ-TTCGCTGCGCTCAACAAGGA-3ʹ (forward) and 5ʹ-TGACAGGGAATCCACAGATGAA-3ʹ (reverse) and 1 × SensiFAST™ SYBR Lo-ROX Kit (BIO-94020, Bioline) in a final volume of 10 µL. Samples were analysed in the QuantStudio 7 Flex Real-Time PCR System (Thermo Fisher Scientific) using the ddCt setting with the cycle program: 95 °C for 20 s and then 40 cycles of 95 °C for 3 s then 60 °C for 30 s, followed by melt curve analysis. Samples were normalised to the expression of 18S and relevant no template controls were included. The quality control of Ct values was based on the Tm and appearance of melt curves and multicomponent plots.

### Cell culture and treatment

Primary astrocyte cultures were derived, cultured and treated as described previously [36]. Briefly, brains from 1- to 3-day-old mice were enzymatically dissociated into mixed glial cell cultures. CD11b-positive cells were removed using the Miltenyi Magnetically-Activated Cell Sorting procedure (Miltenyi) to yield astrocyte cultures. For treatment, hyper-IL-6 [37] was used at 50 ng/mL. To determine the stability of transcripts, cells were treated with 50 ng/mL hyper-IL-6 for 2 h then with actinomycin D (10 µg/mL) (Sigma-Aldrich) or the equivalent volume of DMSO as a vehicle control.

### RNase protection assay

RNase protection assay (RPA) and generation of probes was performed as previously described [38]. Probes used in this study, their NCBI accession number and target regions were: *Sbno2*, NM_183426.1, 800-1149; *Fos*, NM_010234.2, 466-677; *Gbp2*, NM_010260.1, 1100-1255; *Icam1*, X16624, 98-398; *Igtp*, NM_018738.4, 1168-1291; *Irgm1*, NM_008326.1, 953-1176; *Irf1*, M21065, 211-326; *Nlrc5*, NM_001033207.3, 5374-5739; Se*rpina3n*, M64086, 1914-2016; *Socs3*, U88328, 361-555; *Timp1*, X04684, 118-366; *L32*, K02061, 61-139.

### Immunoblotting

Immunoblotting was performed as described previously [27] using the following antibodies and dilutions: SBNO2 (C-terminal #2, custom-made, validated in [27]), 1:640, Biomatik; GAPDH, 1:100,000, Sigma-Aldrich G8795; ERK1/2, 1:10,000, Sigma-Aldrich M5670; pT202-ERK1/pY204-ERK2, 1:5,000, Cell Signaling Technology (CST) 9101; STAT1, 1:1,000, CST 9172; pY701-STAT1, 1:1,000, CST 9167; STAT3, 1:2,000, CST 4904; pY705-STAT3, 1:2,000, CST 9131; pS536-NF-kB, 1:1,000, CST 3033; NF-kB, 1:3,000, CST 8242; Rabbit IgG-peroxidase, 1:30,000, Santa Cruz SC2004; Mouse IgG-peroxidase, 1:10,000, Sigma-Aldrich A0168.

### Microarray and gene ontology enrichment analysis

Cerebella were homogenised in TRI Reagent (Sigma-Aldrich) and RNA was purified using the Direct-zol RNA MiniPrep kit according to the manufacturer’s instructions (Zymo Research). Purified RNA was dissolved in sterile, RNase-free water and purity and concentration of RNA were assessed. Samples were processed by the Ramaciotti Centre for Genomics (University of New South Wales, Sydney, Australia) for cRNA preparation, hybridisation and scanning of Affymetrix murine Clariom S microarrays according to the manufacturer’s instructions (ThermoFisher Scientific). Transcriptome Analysis Console software (version 4.0.1.36, ThermoFisher Scientific) was used to perform gene-level normalisation and summarisation via the in-built Signal Space Transformation-Robust Multi-Chip Analysis algorithm [39, 40]. The Mouse Genome Informatics Gene Expression Database [41] was used to select a background signal value based on the expression of 195 genes deemed not to be expressed in the murine cerebellum, as well as the expression of 219 Y chromosome genes in female samples. A signal intensity threshold of 6 (log2) was found to be a robust cut off and so genes with a signal below 6 were considered to be either not expressed or below the detection limit of the assay. As sex was not controlled for in this study, × and Y chromosome genes were excluded from the analysis. Genes were considered to be differentially regulated between two genotypes if they had a statistically significant (false discovery rate (FDR)-adjusted *p* value < 0.05) fold change of ± 2. Gene ontology (GO) overrepresentation was performed using the PANTHER Overrepresentation Test (Released 20190711; annotation version: GO Ontology database (Biological Process) Released 2019-12-09; Test Type: Fisher's Exact; Correction: FDR) [42]. The background population was defined as genes from the microarray which was expressed (i.e. ≥ 6) in any one genotype. Probes that could not distinguish multiple gene products (denoted by a semicolon in the gene lists) were excluded from the analysis.

### Statistical analysis

Statistical analyses were performed as follows unless otherwise stated. Analyses and graphing were performed using GraphPad Prism software (version 8). The data were compared by two-way ANOVA with Tukey's post test and presented as the mean ± SEM. Differences were considered significant at *p* < 0.05. The number of samples analysed is given in the figure legends. Detailed statistics are presented in Additional file 11.

## Results

### *Sbno2*^*−/−*^ mice are smaller but otherwise overtly normal

**Fig. 2:**
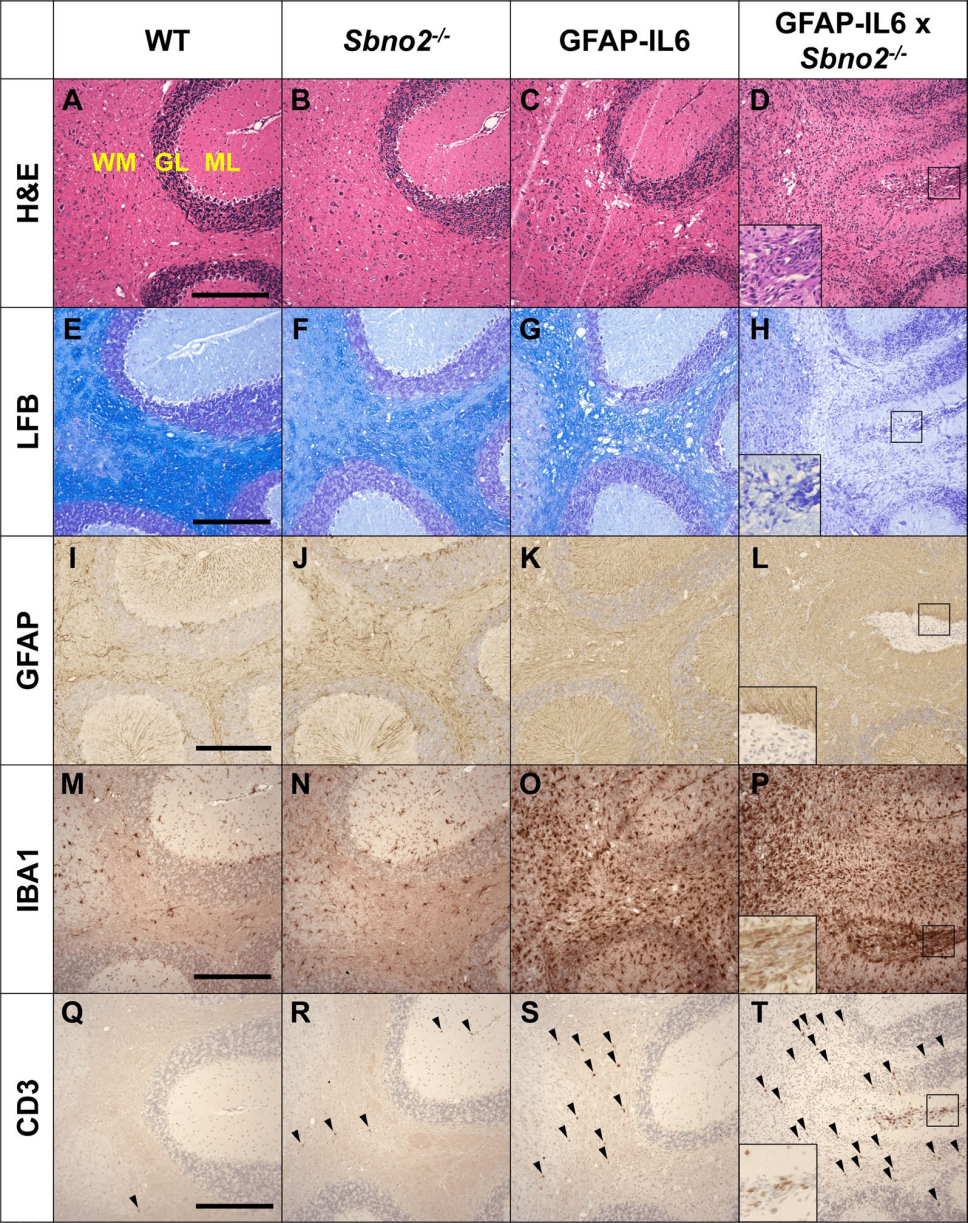
Histochemistry and immunohistochemistry of the cerebellum at 7 months of age. Histochemistry (**A**-**D**, hematoxylin and eosin, H&E; **E**–**H**, luxol fast blue, LFB, for myelin) and immunohistochemistry (**I**–**L**, GFAP for astrocytes; **M**–**P**, IBA1 for microglia; **Q**–**T**, CD3 for T-cells; arrowheads) was performed on paraffin embedded brain sections from WT, *Sbno2*^*−/−*^, GFAP-IL6 and GFAP-IL6 × *Sbno2*^*−/−*^ mice (*n* = 8 per genotype). Pictured is the white matter (WM), granular layer (GM) and molecular layer (ML). CD3+ cells in sulci not annotated with arrowheads for clarity. Representative images shown. Scale bar represents 200 μm. One representative image of n = 8 mice per genotype is shown

To study the role of SBNO2 in the CNS, we first generated mice in which *Sbno2* was disrupted (*Sbno2*^*−/−*^) (Fig. 1A). The recombination of *Sbno2* exons 8 to 10 was confirmed in both DNA and RNA samples (Fig. 1B, C). Although *Sbno2* transcript upstream of the recombined region was present, no SBNO2 protein was detected using an antibody that targets a region downstream of the recombination site (Additional file 1). Breeding male *Sbno2*^*−/−*^ with female *Sbno2*^±^ mice resulted in offspring that were viable and suckled to weaning age, however, we found an underrepresentation of female offspring (40% female versus 60% male) while obtaining genotypes in the expected Mendelian ratios. Adult *Sbno2*^*−/−*^ mice showed a slight decrease in body weight compared with *Sbno2*^+*/*+^ (wild type, WT) mice (Fig. 1D), but were otherwise physically normal and showed no signs of disease (Fig. 1F). Histological analysis of peripheral organs (kidney, liver, spleen, small intestine) and the CNS (brain and spinal cord) revealed no overt abnormalities in adult *Sbno2*^*−/−*^ mice compared with WT mice (Fig. 2 and data not shown).

### SBNO2 protects mice from IL-6-induced neurological disease

To assess the contribution of SBNO2 to IL-6-mediated neuroinflammation in vivo*,* we crossed the *Sbno2*^*−/−*^ mouse with the GFAP-IL6 mouse model. At 1 month of age, both the GFAP-IL6 mice and the GFAP-IL6 × *Sbno2*^*−/−*^ mice were physically indistinguishable from WT mice. However, GFAP-IL6 and GFAP-IL6 × *Sbno2*^*−/−*^ mice had progressively diminished body weight compared with WT and *Sbno2*^*−/−*^ mice and body weight was significantly lower in GFAP-IL6 × *Sbno2*^*−/−*^ mice compared with GFAP-IL6 mice at 3 and 7 months of age (Fig. 1D). However, IL-6 levels were relatively unchanged, being slightly, but not significantly decreased in GFAP-IL6 × *Sbno2*^*−/−*^ mice compared with GFAP-IL6 mice at 1 and 3 months of age (Fig. 1G). Transgene expression in the GFAP-IL6 brain is highest in the cerebellum [20]. Accordingly, GFAP-IL6 mice progressively develop mild ataxia from around 2 months of age, which at 7 months presents as mild splaying of the back feet with an otherwise normal gait (Fig. 1E). In contrast, GFAP-IL6 × *Sbno2*^*−/−*^ mice quickly developed overt ataxia and by 7 months nearly all mice were affected, exhibiting a wobbly gait with occasional falls. The age at onset of the disease was comparable between both genotypes.

Histological and immunohistochemical analysis (Fig. 2) of the cerebellum of 7 month-old GFAP-IL6 mice showed a vacuolated appearance in the cerebellar white matter apparent in both H&E- (Fig. 2C) and LFB-stained sections (Fig. 2G), in line with previous reports [16, 18, 19, 43]. This was accompanied by a loss of myelin, which was most apparent at the outer branches of the cerebellar *arbor vitae*, gliosis of the Bergmann glia in the molecular layer with thickened processes (Fig. 2K), hypertrophy of microglia with stunted processes (Fig. 2O), meningeal leukocyte infiltrates and a diffuse infiltration of the brain’s parenchyma by CD3-positive cells (Fig. 2S). The pathology in the GFAP-IL6 × *Sbno2*^*−/−*^ cerebellum was more severe when compared with the GFAP-IL6 cerebellum. H&E staining revealed a near complete loss of architecture at the centre of the cerebellum (Fig. 2D), with the granule cell layer having degraded to the point of being indistinguishable and the molecular layer being thinned. LFB staining showed a near complete loss of myelin in the white matter in the cerebellum of GFAP-IL6 × *Sbno2*^*−/−*^ mice (Fig. 2H). GFAP immunohistochemistry appeared diffuse and disorganised, with individual astrocytes nearly impossible to distinguish and the GFAP-stained radial processes of the Bergman glia were not distinguishable in areas where the granular layer had degraded (Fig. 2L), although GFAP DAB intensity was not significantly different between the two genotypes (Additional file 2). IBA1 immunohistochemistry, a marker for microglia and monocytes/macrophages, was significantly increased throughout the GFAP-IL6 × *Sbno2*^*−/−*^ cerebellar white matter only (Fig. 2P; Additional file 2). Sulci contained a large number of infiltrating cells, many of which were IBA1- or CD3-positive indicating the presence of monocytes/macrophages and T cells (Fig. 2P, T). The infiltration of CD3-positive T-cells throughout the cerebellum was significantly increased in GFAP-IL6 × *Sbno2*^*−/−*^ compared with that in the GFAP-IL6 cerebellum (Fig. 2T; Additional file 2).

### Exacerbated pathology is paralleled by enhanced IL-6-responsive gene expression.

**Fig. 3:**
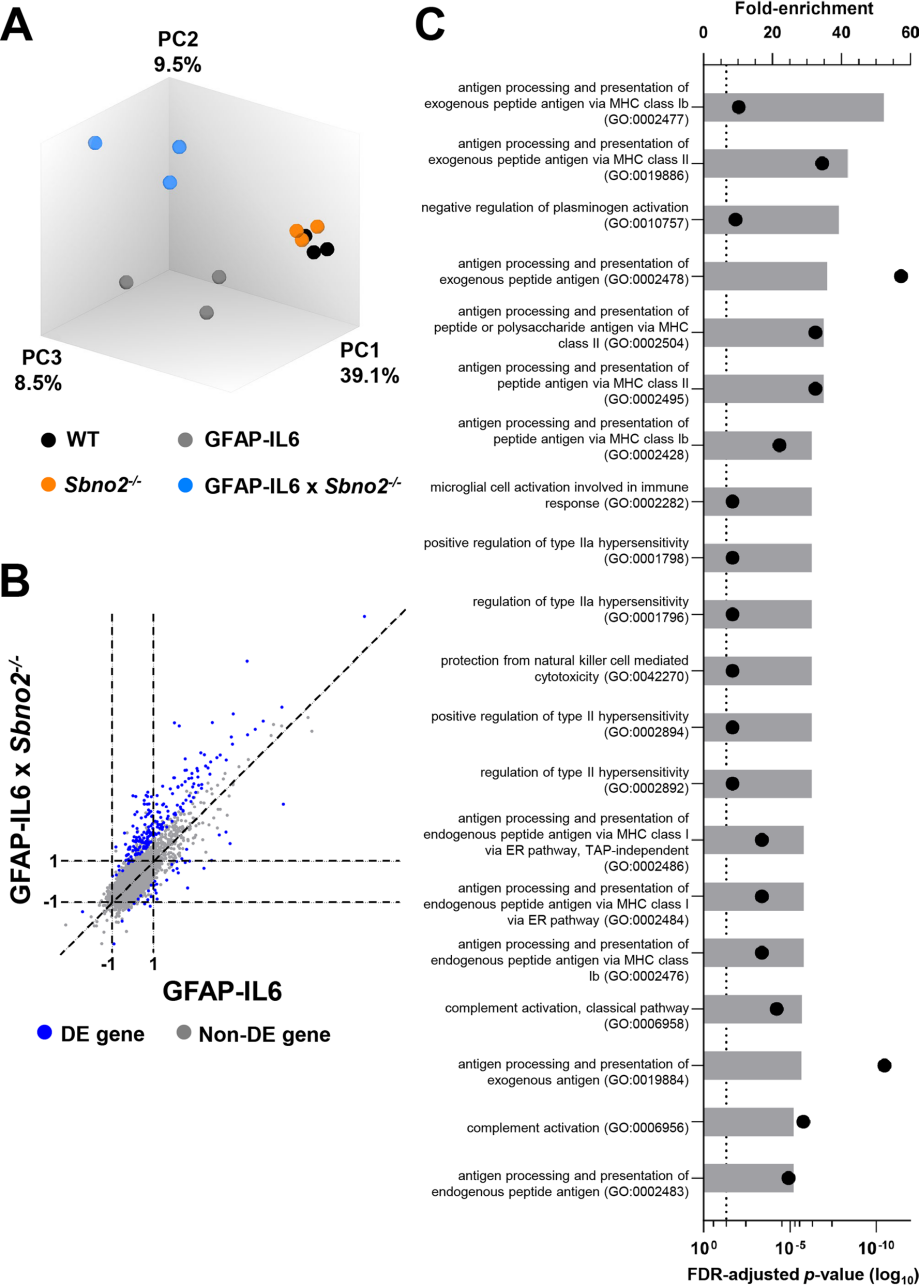
Differential expression analysis and gene ontology overrepresentation tests. **A** Principal component analysis. Each dot represents a sample. The values in the axis labels represent proportion of overall variance as described by each principal component, with PC1 describing the predominant amount of variance (39.1%). **B** Scatter plot of differential gene regulation. Each dot represents a gene expressed in at least WT, GFAP-IL6 or GFAP-IL6 × *Sbno2*^*−/−*^. The *x* axis represents the fold-change (log_2_) of gene expression in GFAP-IL6 when compared with in WT, the *y* axis represents the fold-change (log_2_) of gene expression in GFAP-IL6 × *Sbno2*^*−/−*^ when compared with in WT. Blue dots (DE gene) are genes differentially expressed (a significant fold change (log_2_) of ≥ 1 or ≤ − 1) between GFAP-IL6 and GFAP-IL6 × *Sbno2*^*−/−*^ mice, grey dots (non-DE gene) are genes which are not. Genes which were equally expressed in both GFAP-IL6 and GFAP-IL6 × *Sbno2*^*−/−*^ when compared with WT fall along the diagonal. **C** Top 20 of 275 biological process gene ontology terms enriched amongst genes upregulated in GFAP-IL6 × *Sbno2*^*−/−*^ mice compared with GFAP-IL6 mice. The top *x* axis (bars) represents fold enrichment, the bottom *x* axis (dots) represents the FDR-adjusted *p* value (log_10_), dotted line represents FDR-adjusted *p* value of 0.05

Strawberry notch family members have been implicated in the regulation of gene expression and we have demonstrated that *Sbno2* is markedly upregulated in response to IL-6 [27]. Therefore, we hypothesised that SBNO2 is a putative feedback transcriptional repressor and that the exacerbated pathology in the GFAP-IL6 × *Sbno2*^*−/−*^ cerebellum was due to an enhanced transcriptional response to IL-6 in the absence of SBNO2. To investigate this, differential gene expression profiling was performed on RNA isolated from the cerebellum of WT, *Sbno2*^*−/−*^, GFAP-IL6 and GFAP-IL6 × *Sbno2*^*−/−*^ mice (Fig. 3). We used 1-month old mice as at this age neuropathology in both GFAP-IL6 and GFAP-IL6 × *Sbno2*^*−/−*^ mice was modest (Additional file 3), allowing us to assess the disruption of *Sbno2* more directly.

Approximately 11,000 genes were expressed in each genotype (Additional file 4). No genes were differentially regulated in *Sbno2*^*−/−*^ when compared with WT mice, which was reflected in the principal component analysis (Fig. 3A). However, 385 genes were upregulated and 37 downregulated in GFAP-IL6 compared with WT mice (Fig. 3B, Additional file 5) and 696 genes were upregulated and 117 downregulated in GFAP-IL6 × *Sbno2*^*−/−*^ compared with WT mice (Fig. 3B, Additional file 6). Further, when comparing GFAP-IL6 and GFAP-IL6 × *Sbno2*^*−/−*^ mice, 217 genes were upregulated and 36 downregulated in GFAP-IL6 × *Sbno2*^*−/−*^ mice (Additional file 7). These findings further support the notion that SBNO2 acts predominantly as a repressor in the context of the transcriptional response to IL-6 in the murine CNS. The findings also suggest that the role of SBNO2 in regulating gene expression in adolescent mice under physiological conditions is, at the best, minor.

To better understand the biological outcomes brought about by differential gene regulation, we used the PANTHER overrepresentation test [44, 45] to identify the ‘biological process’ gene ontology (GO) terms overrepresented in each of the gene lists. There were no overrepresented GO terms amongst genes downregulated in any genotype comparison. Amongst genes upregulated in GFAP-IL6 mice compared with WT mice, 616 GO terms were enriched (Additional file 8), most of which related to the immune system as reflected in the most significantly enriched GO term being ‘immune system process’ (GO:0002376). Further, many GO terms related to phenotypes of the GFAP-IL6 model which have previously been experimentally verified, such as microglial cell activation [18, 46], endothelial involvement and angiogenesis [16, 19], cell adhesion [47], chemotaxis [20], complement [43] and the acute-phase response [16, 48]. Amongst genes upregulated in GFAP-IL6 × *Sbno2*^*−/−*^ mice compared with WT mice, 817 GO terms were enriched (Additional file 9), 68% of which were also enriched in GFAP-IL6. Terms highly enriched in both analyses included those relating to hypersensitivity (largely due to Fc receptor genes), complement and ‘microglial cell activation involved in immune response’. Notably, terms relating to MHC molecules were highly enriched in GFAP-IL6 × *Sbno2*^*−/−*^, specifically. These results were re-enforced by examining the 275 GO terms enriched amongst genes upregulated in GFAP-IL6 × *Sbno2*^*−/−*^ compared with GFAP-IL6, in which the MHC-related terms were the most highly enriched (Fig. 3C, Additional file 10). These findings suggest that many of the biological processes which underlie the pathology of the GFAP-IL6 brain also underlie that in the GFAP-IL6 × *Sbno2*^*−/−*^ brain, but furthermore, that some biological processes may be unique to the GFAP-IL6 × *Sbno2*^*−/−*^ model.

### SBNO2 modulates IL-6 stimulated gp130 pathway activation in vivo

**Fig. 4:**
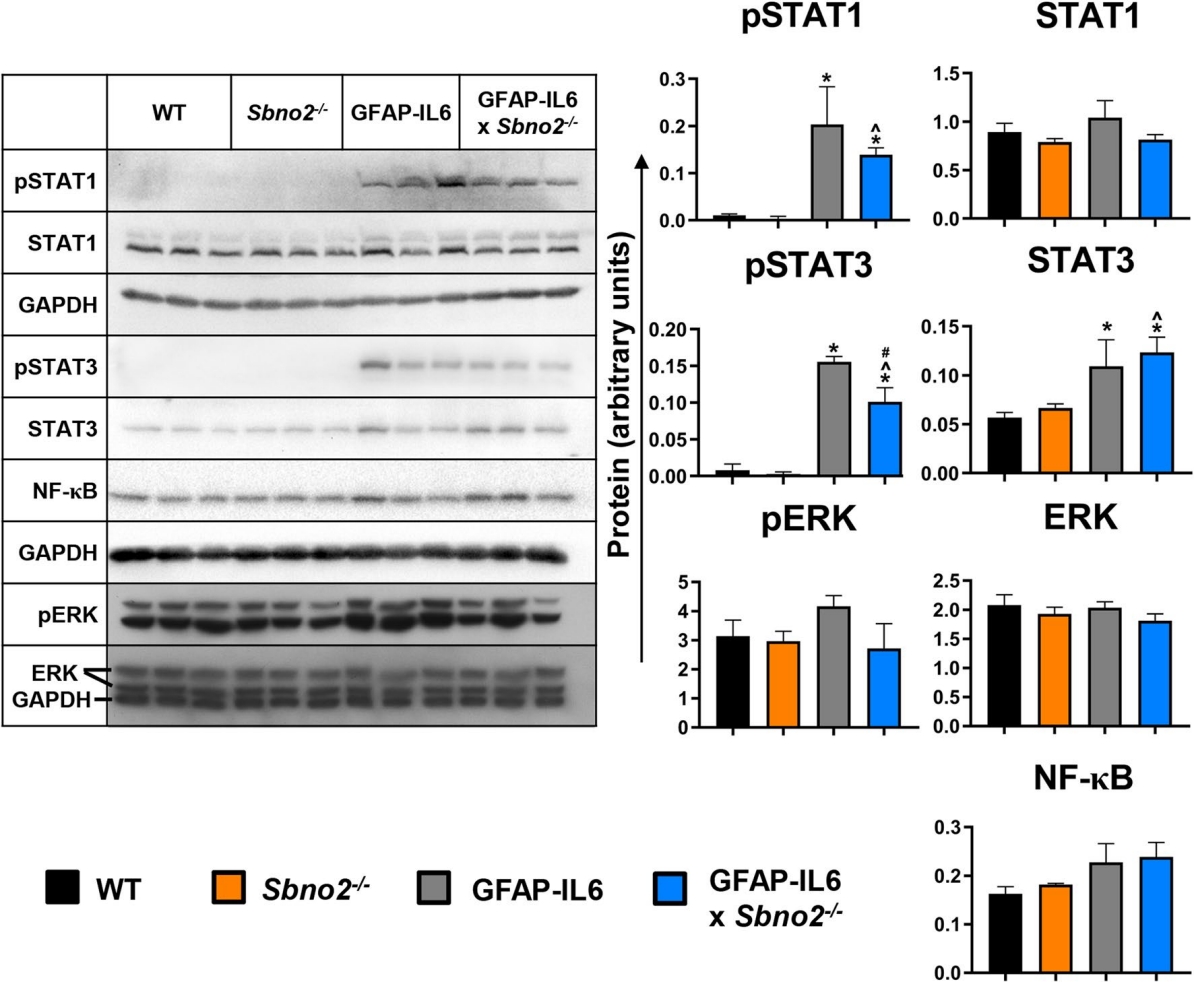
Immunoblot using cerebellar lysates from 1 month old mice. WT, *Sbno2*^*−/−*^, GFAP-IL6 and GFAP-IL6 × *Sbno2*^*−/−*^ mice (*n* = 3 for each genotype). pNF-kB was not detected. Values from densitometric analysis were normalized to the corresponding GAPDH loading control. *, *p* < 0.05 compared with WT; ^, *p* < 0.05 compared with *Sbno2*^*−/−*^; #, *p* < 0.05 compared with GFAP-IL6

Noting that the phenotype of the GFAP-IL6 × *Sbno2*^*−/−*^ brain appeared to be largely an exacerbation of the response to IL-6, yet IL-6 itself was not increased (Fig. 1G), we sought to clarify whether there were changes in the activation of the canonical IL-6/gp130 signalling pathways that might account for this. For this, we examined the key IL-6-responsive gp130 signalling components, STAT1, STAT3 and ERK, as well as the potential SBNO2-interactor, NF-κB [28] in cerebellar lysates from 1 month-old mice (Fig. 4). In lysates from WT and *Sbno2*^*−/−*^ mice, phospho-(p)STAT1 and pSTAT3 were undetectable, while pERK was present and comparable between both genotypes. Lysates from GFAP-IL6 mice showed pSTAT1 and pSTAT3 signals, while pERK was comparable to WT and *Sbno2*^*−/−*^ mice. In GFAP-IL6 × *Sbno2*^*−/−*^ mice, levels of pSTAT3 were significantly reduced when compared with GFAP-IL6 mice, while pSTAT1 and pERK levels were lower, albeit not significantly. Phosphorylation of NF-κB was undetectable in all genotypes. These findings show there is diminished IL-6-stimulated gp130 pathway activation in the GFAP-IL6 × *Sbno2*^*−/−*^ cerebellum.

### SBNO2 acts as a negative regulator of IL-6-stimulated gene expression

**Fig. 5:**
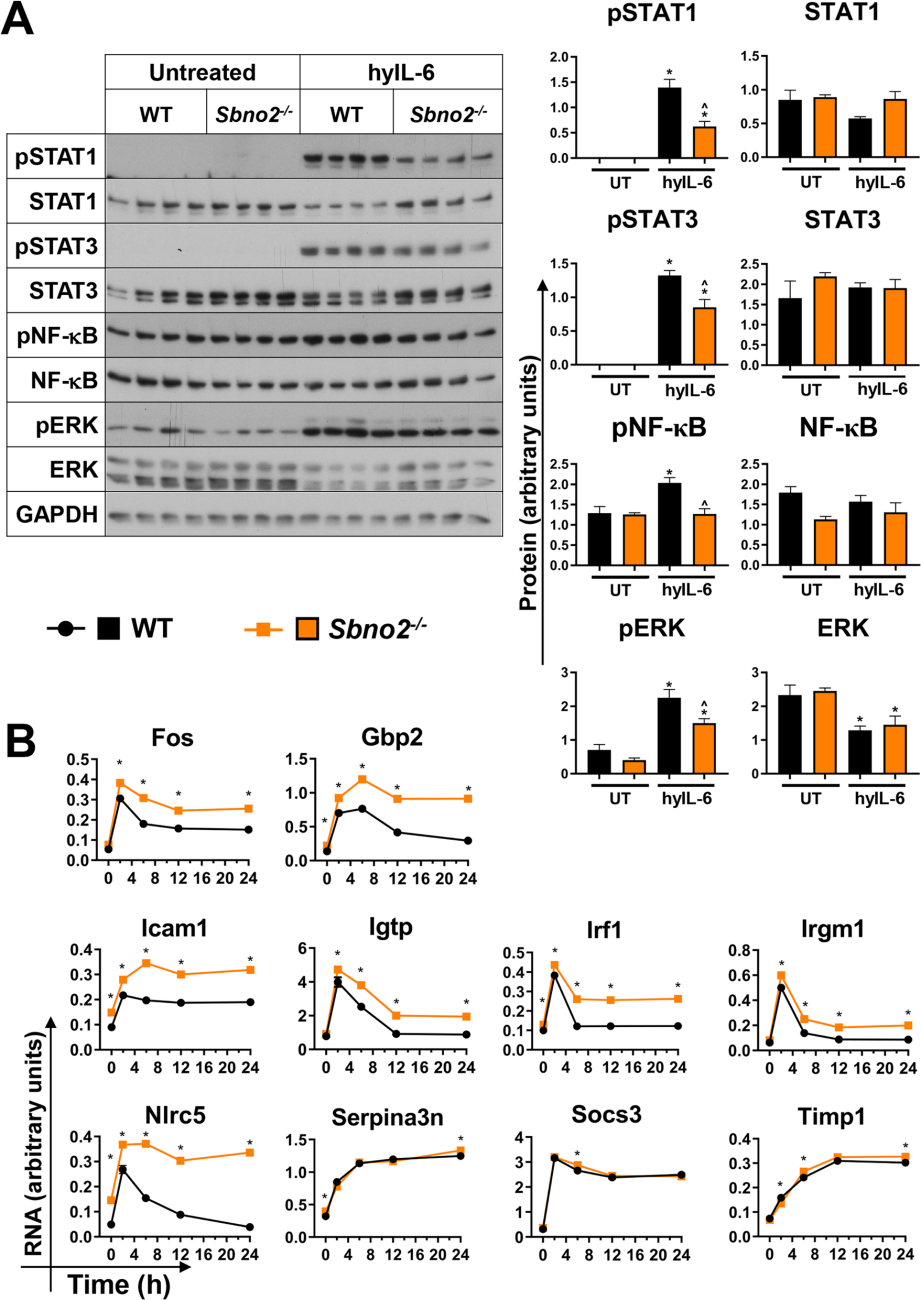
Immunoblot and RPA using hyper-IL-6-treated primary cultured astrocytes. **A** Decreased signal pathway phosphorylation in primary astrocytes treated for 6 h with hyper-IL-6. Immunoblot was performed on protein lysates from WT and Sbno2−/− primary cultured astrocytes untreated (UT) or treated with hyper-IL-6 (hyIL-6) for 6 h to determine the levels of given proteins (n = 4 for each genotype). Values from densitometric analysis were normalized to the corresponding GAPDH loading control. **B **RPA was performed on total RNA from WT and *Sbno2*^*−/−*^ primary cultured astrocytes to determine the level of RNA transcript for given genes (*n* = 3 for each genotype). * *p* < 0.05 between genotypes at each time point. *, *p* < 0.05 compared with untreated of same genotype; ^, *p* < 0.05 compared with corresponding WT

Decreased phosphosignalling in the GFAP-IL6 × *Sbno2*^*−/−*^ cerebellum suggested that SBNO2 may alter IL-6/gp130 signal pathway activation. In addition, as SBNO2 production plateaued after 6 h of hyper-IL-6 treatment in WT astrocytes [27], we asked whether sustained gene expression in hyper-IL-6-treated *Sbno2*^*−/−*^ astrocytes may reflect a need for SBNO2 to repress excessive signal pathway activation. Therefore, we treated *Sbno2*^*−/−*^ astrocytes with hyper-IL-6 for 6 h and examined IL-6/gp130 signalling pathway components STAT1, STAT3, ERK as well as NF-κB (Fig. 5A). A two-way ANOVA was performed to analyse the effect of *Sbno2* disruption and of hyper-IL-6 treatment on protein levels (Additional file 11). The main effects analysis showed that the treatment had a statistically significant effect on pSTAT1, pSTAT3, pERK, ERK and pNF-κB. *Sbno2* disruption had a statistically significant effect on pSTAT1, pSTAT3, pERK, pNF-κB and NF-κB. There was a statistically significant interaction between these effects on pSTAT1, pSTAT3 and pNF-κB. In both WT and *Sbno2*^*−/−*^ astrocytes, hyper-IL-6 treatment increased pSTAT1, pSTAT3 and pERK levels, but to a significantly lesser degree in *Sbno2*^*−/−*^ cells. The pNF-κB level increased significantly with treatment in WT, but not *Sbno2*^*−/−*^ cells. These results reveal that disruption to *Sbno2* causes decreased levels of hyper-IL6-stimulated gp130 pathway activation in primary cultured astrocytes.

The information gleaned from the GFAP-IL6 × *Sbno2*^*−/−*^ model provided a chronic, in vivo view of SBNO2-modulated gene expression. Next, we wished to delineate the cellular function of SBNO2 and how it affected the temporal response to an acute exposure to IL-6. We had previously shown that *Sbno2* is predominantly, but not exclusively, expressed by astrocytes in the CNS [27]. Therefore, we treated primary cultured astrocytes from WT and *Sbno2*^*−/−*^ mice with hyper-IL-6 and examined the expression of several genes associated with CNS inflammation over 24 h (Fig. 5B). A two-way ANOVA was performed to analyse the effect of *Sbno2* disruption and of hyper-IL-6 treatment time on gene expression (Additional file 11). Main effects analysis showed that treatment time had a statistically significant effect on the expression of all genes examined, and that *Sbno2* disruption had a statistically significant effect on *Fos, Gbp2, Icam1, Igtp, Irf1, Irgm1, Nlrc5* and *Socs3*. There was a statistically significant interaction between the effects on all genes besides *Socs3*. The expression of *Gbp2*, *Icam1*, *Irf1*, *Nlrc5* and *Serpina3n* was significantly higher in untreated *Sbno2*^*−/−*^ cells than in untreated WT cells, suggesting some requirement for SBNO2 to maintain basal expression of these genes in vitro. The expression of *Fos, Gbp2, Icam1, Igtp, Irf1, Irgm1* and *Nlrc5* was significantly higher in *Sbno2*^*−/−*^ cells than in WT cells following hyper-IL-6 treatment (at 2 h, 6 h, 12 h and 24 h), while expression of *Serpina3n*, *Socs3* and *Timp1* was significantly higher only at certain times. Expression of *Igtp, Irf1, Irgm1* and *Nlrc5* returned to their basal level of expression in WT cells, but levels were sustained in *Sbno2*^*−/−*^ cells. These results suggest that SBNO2 is required to actively dampen expression of a subset of genes as part of the negative regulation of the IL-6 transcriptional response.

### Enhanced gene expression in *Sbno2*^*−/−*^ astrocytes is not due to differences in gene transcript stability

**Fig. 6:**
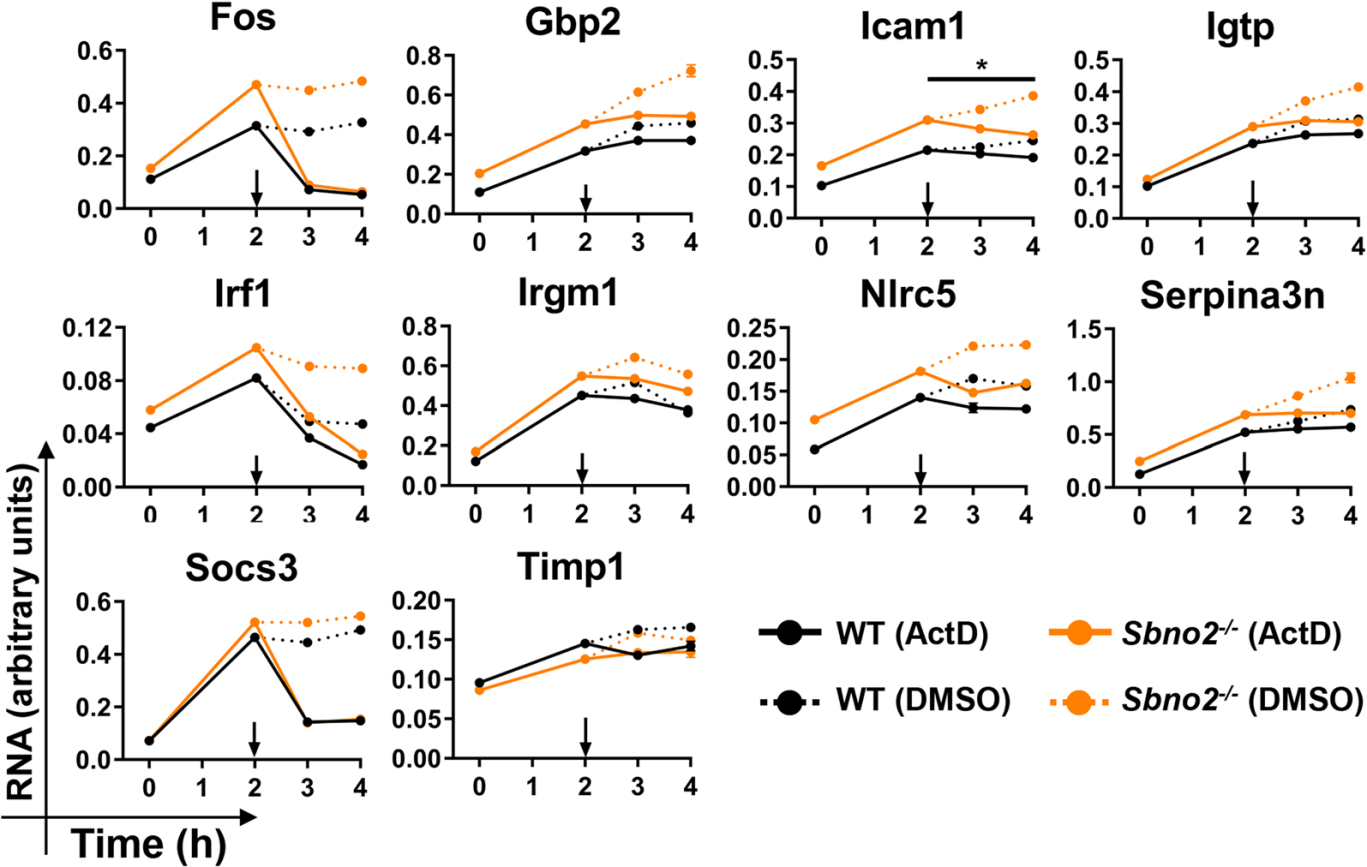
RPA using primary cultured astrocytes treated with hyper-IL-6-treated and actinomycin D (ActD). WT and *Sbno2*^*−/−*^ primary cultured astrocytes were treated with hyper-IL-6 for 2 h then treated with ActD (arrow) which inhibited transcription to determine transcript stability. RPA was performed on total RNA to determine the level of RNA transcript for given genes (*n* = 3). DMSO was used as a vehicle. Linear regression was performed using ActD values between 2 and 4 h and comparing genotypes using ANCOVA analysis [70, 71]. *, *p* < 0.05 between genotypes

Having determined that increased signal pathway activation was not responsible for sustained gene expression in *Sbno2*^*−/−*^ astrocytes, we next sought to address whether changes in gene transcript stability played a role. To examine this, *Sbno2*^*−/−*^ primary cultured astrocytes were treated with hyper-IL-6 for 2 h to stimulate gene expression, then actinomycin D (ActD) was added, which inhibited further transcription, revealing transcript stability over the following 2 h. No differences between *Sbno2*^*−/−*^ and WT cells were found for *Fos, Gbp2, Icam1, Igtp, Irf1, Irgm1, Nlrc5, Serpina3n* and *Socs3* transcripts, while the *Icam1* transcript was found to be significantly different (Fig. 6). Overall, these results suggest that sustained gene expression in *Sbno2*^*−/−*^ astrocytes is not elicited through SBNO2 modulating transcript stability.

## Discussion

The strawberry notch family of nuclear, putative helicases are emerging regulators of gene expression. Members of this conserved family have been described in *Arabidopsis, Caenorhabditis elegans, Drosophila melanogaster, Danio rerio, Mus musculus*, and *Homo sapiens*, yet relatively little is known about their role or function. We have previously shown that in the murine CNS, *Sbno2* is expressed predominantly by astrocytes [27]. Further, expression of *Sbno2* is tightly-regulated and is highly responsive to stimulation by gp130 family cytokines, including IL-6. Here, we demonstrate that SBNO2 is required to constrain IL-6-driven neurological disease. This is underpinned by exaggerated pathology and increased gene expression in the cerebellum of GFAP-IL6 × *Sbno2*^*−/−*^ mice. Using in vitro studies, we show that the transcriptional response to IL-6 is heightened and prolonged in *Sbno2*^*−/−*^ astrocytes. Ultimately, our findings demonstrate that SBNO2 is a novel regulator providing negative feedback to IL-6-driven gene expression.

Apart from a slight decrease in the body weight, we observed no overt abnormalities in *Sbno2*^*−/−*^ mice, indicating minor roles for SBNO2 during physiological conditions. This differs from other strawberry notch family homologs, including murine SBNO1, which have important or critical roles in development [24, 49]. However, our findings support those by Maruyama et al*.* [25] that found *Sbno2*^*−/−*^ mice to be viable. In contrast, El Kasmi et al*.* [28] reported that *Sbno2*^*−/−*^ mice die at the early embryonic stage. The reason for this discrepancy with the findings by El Kasmi et al. is unclear but may be due to differences in the loci targeted in the respective knockout approaches. A truncated protein may exist in Maruyama et al*.*, given that they target exons 19 to 25 for recombination, and in our own, given that the 5’ end of the *Sbno2* transcript was detected. If true, this might suggest an essential role for the N-terminus of SBNO2. However, as no SBNO2 protein was detected using a C-terminal-reactive antibody, we anticipate that the putative helicase domains, and therefore, predicted regulatory function of SBNO2 is not present in our model.

**Fig. 7:**
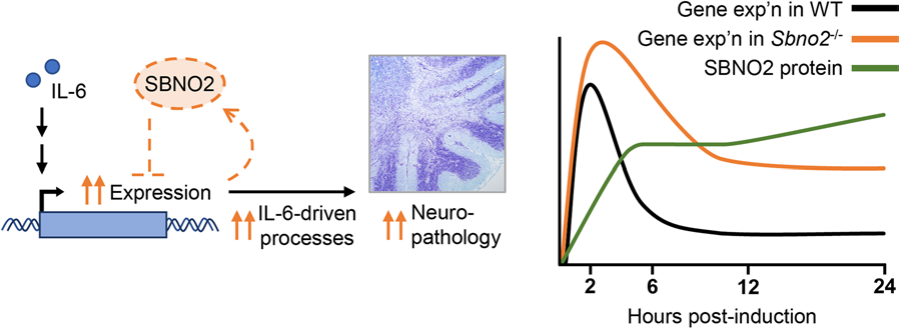
Model for SBNO2 as a negative feedback regulator in the transcriptional response to IL-6. In WT cells, IL-6 stimulates the expression of certain genes (black line, right) including *Sbno2*. The production of SBNO2 protein (green line, right) contributes to the reduction in expression of some of these genes post-stimulation. In *Sbno2*^*−/−*^ cells, the absence of SBNO2 (dashed outline, left) results in heightened and prolonged expression (orange line, right), increasing the intensity of associated IL-6-driven biological processes and, in the GFAP-IL6 × *Sbno2*^*−/−*^ cerebellum, subsequent neuropathology (left). Temporal regulation of SBNO2 protein adapted from [27]

In contrast to physiological conditions, the absence of SBNO2 in the GFAP-IL6 transgenic mice exacerbated the progressive cerebellar neurodegeneration characteristic of this model, resulting in increased inflammatory gene expression, greater destructive pathology and more severe clinical symptoms. These findings suggest that SBNO2 is a novel regulator for providing negative feedback in the transcriptional response to IL-6 and represses the detrimental effects of IL-6 in the CNS (Fig. 7). This is further supported by our finding that *Sbno2*^*−/−*^ resulted in the heightened and prolonged expression of some IL-6-stimulated genes in vitro. It is interesting to note that known mechanisms for negative regulation of the intracellular IL-6 signal either are non-nuclear-localised and their production is induced by IL-6 (such as the suppressor of cytokine signalling (SOCS) proteins [50–53]), or conversely, are nuclear-localised and their production is not induced by IL-6 (such as the protein inhibitor of activated STAT (PIAS) proteins [54, 55], histone deacetylase 1 (HDAC1) [56, 57] and peroxisome proliferator-activated receptor gamma (PPAR-γ) [58–61]). Thus, our findings identify SBNO2 as the first regulator of IL-6 signalling which is both nuclear-localised and whose production is induced by IL-6.

Heightened gene expression in *Sbno2*^*−/−*^ mice was not due to increased IL-6 cytokine levels, increased IL-6 signal pathway activation or increased transcript stability. In contrast, our findings show reduced activation of the canonical JAK-STAT signalling pathway in the absence of SBNO2. Rather, as SBNO2 belongs to a family of nuclear proteins, we propose that SBNO2 exerts its effects at the DNA–protein interface. SBNO2 has no clear sequence-specific DNA-binding domain and so its target gene specificity might be conferred by the transcription factors it acts upon. Since other strawberry notch family members modulate the dissociation of transcriptional regulators from the genome [24–26], we speculate that murine SBNO2 acts by removing activating transcription factors to dampen the expression of IL-6-stimulated genes. In this scenario, transcription factors lingering on promoters in the absence of SBNO2 might account for the counterintuitive heightened gene expression in the absence of increased signal pathway activation. Indeed, the regulated step of STAT1 inactivation is its dissociation from the promoter and not tyrosine-701 dephosphorylation [62]. It is interesting to note that prototypical STAT3 regulated genes—*Serpina3n*, *Socs3*, *Timp1—*were minimally affected by the loss of Sbno2. The reason underlying this difference in STAT1- vs. STAT3-regulated genes remains unclear.

Transcriptomic analysis provided insight into potential mechanisms of IL6-induced inflammation and neurodegeneration. GO terms relating to the complement system and microglia were among the most highly enriched in the GFAP-IL6 and GFAP-IL6 × *Sbno2*^*−/−*^ analyses. The production of complement and microglial activation have been noted previously in the GFAP-IL6 brain [18, 43]. More recently, complement proteins have been found to mark neuronal synapses for destruction by reactive microglia [63, 64]. This process occurs during development, where it prunes excessive and weak synapses, as well as in neurodegenerative processes. In contrast, terms relating to MHC complexes (arising primarily from a number of upregulated MHC-I genes) were highly enriched in the GFAP-IL6 × *Sbno2*^*−/−*^, but not GFAP-IL-6, analysis. Class I MHC molecules, too, have roles in developmental synapse stripping [65] and emerging associations with neurodegeneration [66–68]. Together, the high enrichment of GO terms relating to complement and MHC-I in the GFAP-IL6 × *Sbno2*^*−/−*^ analysis suggests that interoperation of these two systems could be a unique driver of neuropathology in this model. Indeed, complement and MHC-I interoperate during developmental retinogeniculate pruning [69] and similar mechanisms may contribute to neurodegeneration in the GFAP-IL6 × *Sbno2*^*−/−*^brain.

## Conclusions

In summary, our findings demonstrate that murine SBNO2 is a regulator of the IL-6 transcriptional response and provides a novel negative feedback mechanism necessary for protecting against severe IL-6-induced neurodegeneration. These findings build upon the first description of SBNO2 as an anti-inflammatory factor and add to the body of knowledge on the emerging family of strawberry notch homologs. Not only may future studies on SBNO2 and its homologs aid in understanding the processes underlying the actions of IL-6, but they have the potential to provide new insights on gene regulation in a broad range of human and non-human diseases and disorders.

## Supplementary Information

**Additional file 1. Fig. S1.** **A** qPCR using primers targeting upstream (5’) of exon 8 was performed on RNA extracted from the cerebellum of WT and Sbno2 /  mice (n = 4). Result is expressed as fold-change in Ct value when compared with the mean Ct value for WT. **B** Immunoblot was performed on protein lysates from WT and Sbno2 /  primary cultured astrocytes untreated (UT) or treated with hyperIL-6 (hyIL-6) for 6 h to determine the levels of given proteins (n = 3). A C-terminal-reactive antibody was used to detect SBNO2 [27]. Arrow denotes 160 kDa.

**Additional file 2: Fig. S2.** **A** Quantification of DAB intensity in three regions of the cerebellum after GFAP- or IBA1-staining was analysed using a randomized block ANOVA with Tukey post-test. **B** Quantification of CD3-positive cells in the cerebellum, normalised to area counted (arbitrary units; n = 8). *, *p* < 0.05 compared with WT; ^, *p* < 0.05 compared with *Sbno2*^*−/−*^; #, *p* < 0.05 compared with GFAP-IL6.

**Additional file 3: Fig. S3.** Histochemistry & immunohistochemistry of the cerebellum at 1 month of age. Histochemistry (**A-D**, hematoxylin and eosin, H&E; **E–H**, luxol fast blue, LFB, for myelin) and immunohistochemistry (**I-L**, GFAP for astrocytes; **M-P**, IBA1 for microglia) was performed on paraffin embedded brain sections from WT, *Sbno2*^*−/−*^, GFAP-IL6 and GFAP-IL6 × *Sbno2*^*−/−*^ mice. Pictured is the white matter (WM), granular layer (GM) and molecular layer (ML). Representative images shown (*n* = 6 per genotype). Scale bar represents 100 μm.

**Additional file 4.** Genes expressed in WT, *Sbno2*^*−/−*^ (KO), GFAP-IL6 (GIL6) and GFAP-IL6 × *Sbno2*^*−/−*^ (EXP) mice cerebella. Shown are gene symbol, gene description, log2 of signal intensities, Affymetrix probe ID and public gene accession numbers. Note that because of the log2 scale, an increase in signal intensity of + 1 is equivalent to a twofold increase and so on. In cases when the microarray could not distinguish between two or more gene products, genes contributing to the signal are separated by a semicolon.

**Additional file 5.** Differentially expressed genes between GFAP-IL6 (GIL6) and WT mice. Shown are gene symbol, gene description, fold-change, false discovery rate (FDR) *p*-value of fold-change, Affymetrix probe ID and public gene accession numbers. In cases when the microarray could not distinguish between two or more gene products, genes contributing to the signal are separated by a semicolon.

**Additional file 6.** Differentially expressed genes between GFAP-IL6 × *Sbno2*^*−/−*^ (EXP) and WT mice. Shown are gene symbol, gene description, fold-change, false discovery rate (FDR) *p*-value of fold-change, Affymetrix probe ID and public gene accession numbers. In cases when the microarray could not distinguish between two or more gene products, genes contributing to the signal are separated by a semicolon.

**Additional file 7.** Differentially expressed genes between GFAP-IL6 × *Sbno2*^*−/−*^ (EXP) and GFAP-IL6 (GIL6) mice. Shown are gene symbol, gene description, fold-change, false discovery rate (FDR) *p*-value of fold-change, Affymetrix probe ID and public gene accession numbers. In cases when the microarray could not distinguish between two or more gene products, genes contributing to the signal are separated by a semicolon.

**Additional file 8.** Biological process (BP) gene ontology (GO) term overrepresentation analysis of genes upregulated in GFAP-IL6 (GIL6) compared with WT. Shown are the BP GO term; the number of genes associated with the term in the background reference list; the number of genes associated with the term in the analysed list; the expected value, the number of genes expected in the analysed list for the term based on the reference list; the fold enrichment of the genes observed in the analysed list over the expected; the raw p-value as determined by Fisher’s exact test; and the false discovery rate as calculated by the Benjamini–Hochberg procedure.

**Additional file 9.** Biological process (BP) gene ontology (GO) term overrepresentation analysis of genes upregulated in GFAP-IL6 × *Sbno2*^*−/−*^ (EXP) compared with WT. Shown are the BP GO term; the number of genes associated with the term in the background reference list; the number of genes associated with the term in the analysed list; the expected value, the number of genes expected in the analysed list for the term based on the reference list; the fold enrichment of the genes observed in the analysed list over the expected; the raw p-value as determined by Fisher’s exact test; and the false discovery rate as calculated by the Benjamini–Hochberg procedure.

**Additional file 10.** Biological process (BP) gene ontology (GO) term overrepresentation analysis of genes upregulated in GFAP-IL6 × *Sbno2*^*−/−*^ (EXP) compared with GFAP-IL6 (GIL6). Shown are the BP GO term; the number of genes associated with the term in the background reference list; the number of genes associated with the term in the analysed list; the expected value, the number of genes expected in the analysed list for the term based on the reference list; the fold enrichment of the genes observed in the analysed list over the expected; the raw p-value as determined by Fisher’s exact test; and the false discovery rate as calculated by the Benjamini–Hochberg procedure.

**Additional file 11.** Detailed results from statistical analyses.

## Data Availability

Differential gene expression analysis and gene ontology term overrepresentation test datasets are included in this published article and its Additional information files. The microarray dataset generated and analysed during the current study is available in the ArrayExpress database at EMBL-EBI under accession number E-MTAB-11169 at https://www.ebi.ac.uk/arrayexpress/experiments/E-MTAB-11169. Other data used and/or analysed during the current study are available from the corresponding author on reasonable request.

## Abbreviations

ActD: Actinomycin D
ANOVA: Analysis of variance
BP: Biological process
CC: Cellular component
CD: Cluster of differentiation
CNS: Central nervous system
CST: Cell Signalling Technology
DAB: 3,3′-Diaminobenzidine
DMSO: Dimethyl sulfoxide
ELISA: Enzyme-linked immunosorbent assay
EXP: GFAP-IL6 × *Sbno2*^*−*^^*/*^^*−*^
FL: Floxed
GAPDH: Glyceraldehyde 3-phosphate dehydrogenase
GFAP: Glial fibrillary acidic protein
GIL6: GFAP-IL6
GO: Gene ontology
gp130: Glycoprotein 130
H&E: Hematoxylin and eosin
IBA1: Ionized calcium binding adaptor molecule 1
IHC: Immunohistochemistry
IL: Interlukin
IL-6R: IL-6 receptor
JAK: Janus kinase
KO: *Sbno2* ^*−*^^*/*^^*−*^
LFB: Luxol fast blue
MAPK: Mitogen-activated protein kinase
MF: Molecular function
MHC-I: Major histocompatibility complex class I
PBS: Phosphate buffered saline
pERK: Phosphorylated ERK
pNFκB: Phosphorylated NFκB
pSTAT: Phosphorylated STAT
RPA: RNase protection assay
sgp130: Soluble gp130 receptor
sIL-6R: Soluble IL-6 receptor
SOCS: Suppressor of cytokine signalling
STAT: Signal transducer and activator of transcription
UT: Untreated
WT: Wild type
