# Supplementary figures and images for: Strawberry notch homolog 2 regulates the response to interleukin-6 in the central nervous system
Source: J Neuroinflammation. 2022 May 27;19:126. doi: 10.1186/s12974-022-02475-1 (PMC9145108; doi:10.1186/s12974-022-02475-1)

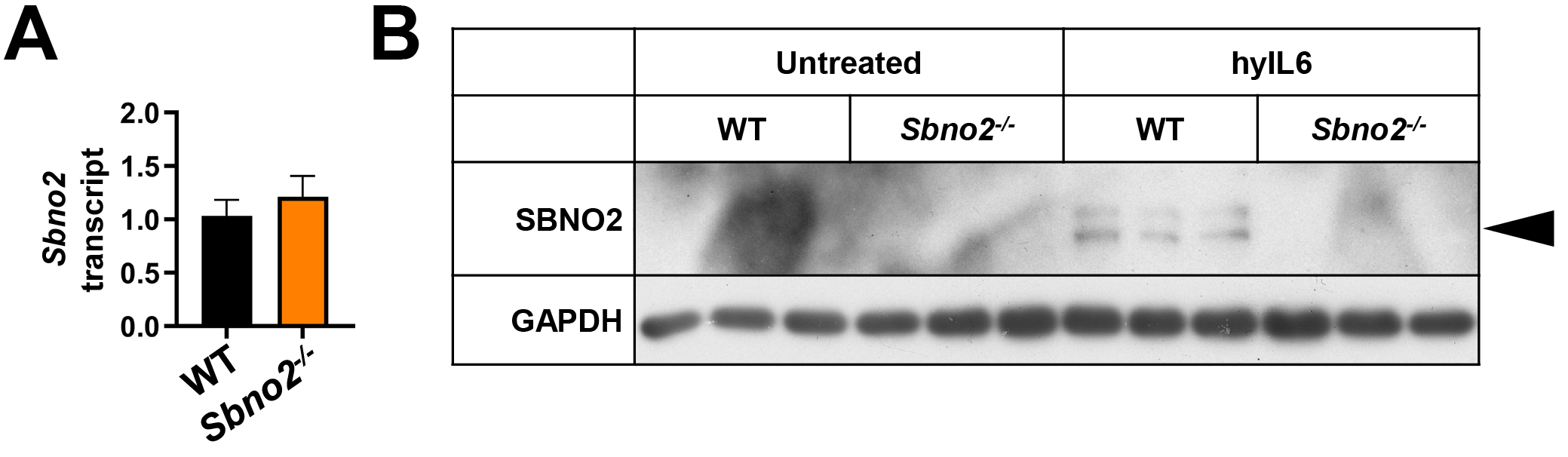

Supplement: Supplementary file 1 — Additional file 1. Fig. S1. A qPCR using primers targeting upstream (5’) of exon 8 was performed on RNA extracted from the cerebellum of WT and Sbno2 / mice (n = 4). Result is expressed as fold-change in Ct value when compared with the mean Ct value for WT. B Immunoblot was performed on protein lysates from WT and Sbno2 / primary cultured astrocytes untreated (UT) or treated with hyperIL-6 (hyIL-6) for 6 h to determine the levels of given proteins (n = 3). A C-terminal-reactive antibody was used to detect SBNO2 [27]. Arrow denotes 160 kDa. [file 12974_2022_2475_MOESM1_ESM.png]

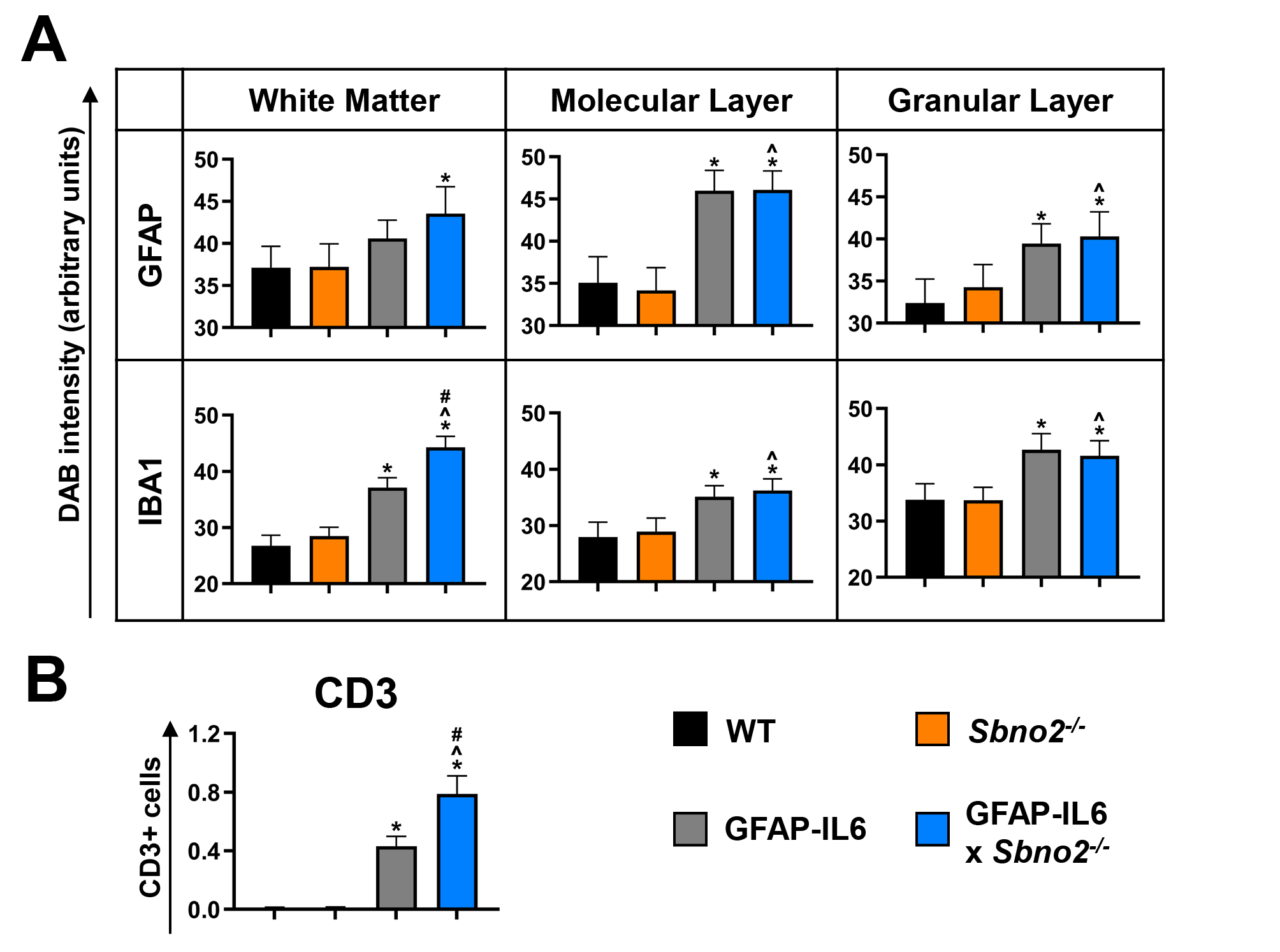

Supplement: Supplementary file 2 — Additional file 2: Fig. S2. A Quantification of DAB intensity in three regions of the cerebellum after GFAP- or IBA1-staining was analysed using a randomized block ANOVA with Tukey post-test. B Quantification of CD3-positive cells in the cerebellum, normalised to area counted (arbitrary units; n = 8). *, p < 0.05 compared with WT; ^, p < 0.05 compared with Sbno2−/−; #, p < 0.05 compared with GFAP-IL6. [file 12974_2022_2475_MOESM2_ESM.png]

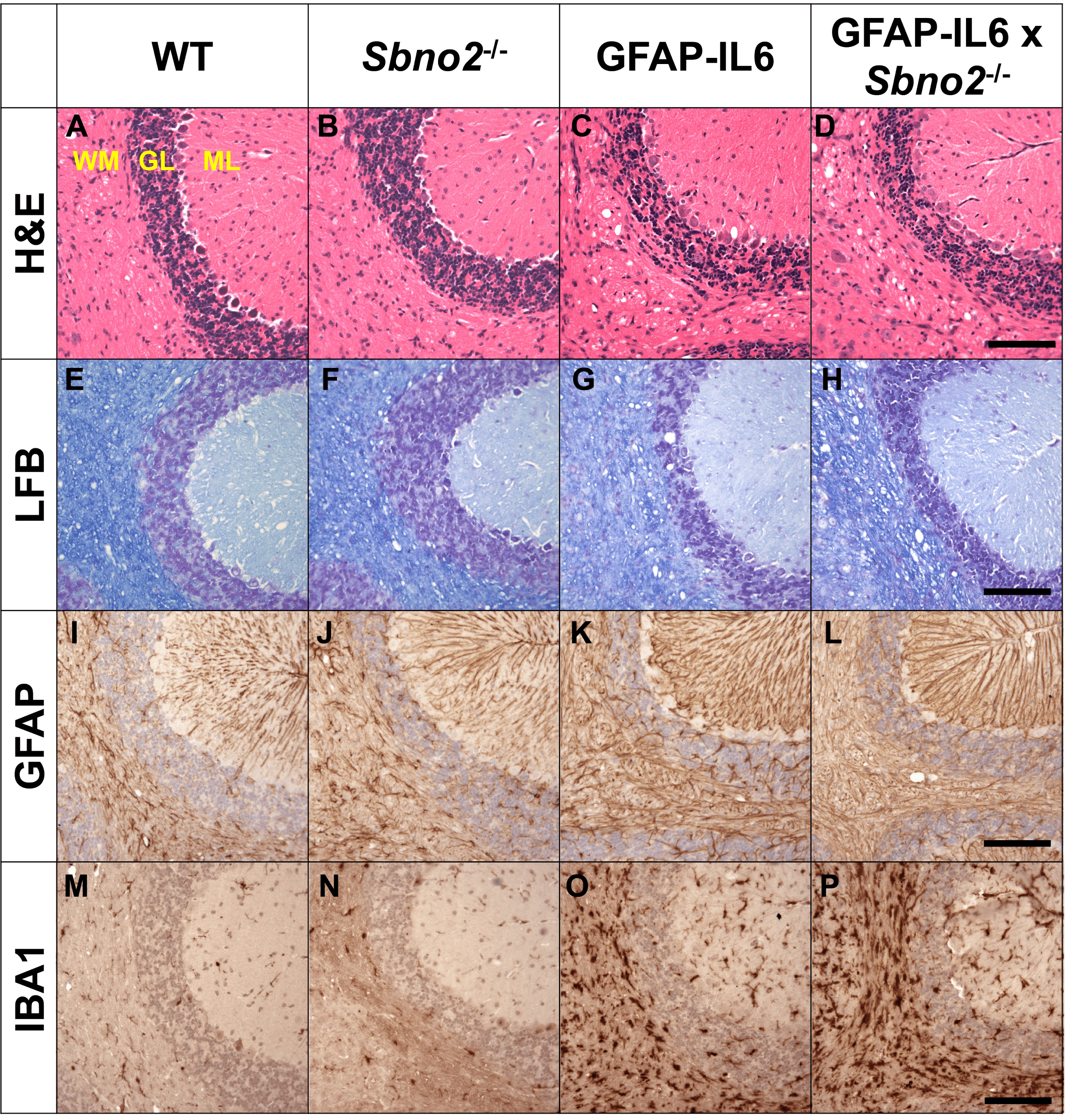

Supplement: Supplementary file 3 — Additional file 3: Fig. S3. Histochemistry & immunohistochemistry of the cerebellum at 1 month of age. Histochemistry (A-D, hematoxylin and eosin, H&E; E–H, luxol fast blue, LFB, for myelin) and immunohistochemistry (I-L, GFAP for astrocytes; M-P, IBA1 for microglia) was performed on paraffin embedded brain sections from WT, Sbno2−/−, GFAP-IL6 and GFAP-IL6 × Sbno2−/− mice. Pictured is the white matter (WM), granular layer (GM) and molecular layer (ML). Representative images shown (n = 6 per genotype). Scale bar represents 100 μm. [file 12974_2022_2475_MOESM3_ESM.png]
